# Near real-time data on the human neutralizing antibody landscape to influenza virus to inform vaccine-strain selection in September 2025

**DOI:** 10.1093/ve/veaf086

**Published:** 2025-11-03

**Authors:** Caroline Kikawa, John Huddleston, Andrea N Loes, Sam A Turner, Jover Lee, Ian G Barr, Benjamin J Cowling, Janet A Englund, Alexander L Greninger, Ruth Harvey, Hideki Hasegawa, Faith Ho, Kirsten Lacombe, Nancy H L Leung, Nicola S Lewis, Heidi Peck, Shinji Watanabe, Derek J Smith, Trevor Bedford, Jesse D Bloom

**Affiliations:** Division of Basic Sciences and Computational Biology Program, Fred Hutch Cancer Center, 1100 Fairview Ave N, Seattle, WA 98109, USA; Department of Genome Sciences, University of Washington, 1959 NE Pacific St, Seattle, WA 98195, USA; Medical Scientist Training Program, University of Washington, 1959 NE Pacific St, Seattle, WA 98195, USA; Vaccine and Infectious Disease Division, Fred Hutch Cancer Center, 1100 Fairview Ave N, Seattle, WA 98109, USA; Division of Basic Sciences and Computational Biology Program, Fred Hutch Cancer Center, 1100 Fairview Ave N, Seattle, WA 98109, USA; Howard Hughes Medical Institute, 1100 Fairview Ave N, Seattle, WA 98109, USA; Centre for Pathogen Evolution, Department of Zoology, University of Cambridge, The Old Schools, Trinity Ln, Cambridge CB2 1TN, United Kingdom; Vaccine and Infectious Disease Division, Fred Hutch Cancer Center, 1100 Fairview Ave N, Seattle, WA 98109, USA; WHO Collaborating Centre for Reference and Research on Influenza, The Peter Doherty Institute for Infection and Immunity, 792 Elizabeth St, Melbourne VIC 3000, Australia; WHO Collaborating Centre for Infectious Disease Epidemiology and Control, School of Public Health, LKS Faculty of Medicine, The University of Hong Kong, Pok Fu Lam, Hong Kong Special Administrative Region; Seattle Children’s Research Institute, 4800 Sand Point Way NE, Seattle, WA 98105, USA; Department of Pediatrics, University of Washington, 4800 Sand Point Way NE, Seattle, WA 98105, USA; Virology Division, Department of Laboratory Medicine and Pathology, University of Washington Medical Center, 1959 NE Pacific St, Seattle, WA 98195 USA; Worldwide Influenza Centre, The Francis Crick Institute, 1 Midland Rd, London NW1 1AT, United Kingdom; Influenza Research Center, National Institute of Infectious Diseases, Japan Institute for Health Security, 1-21-1 Toyama Shinjuku-ku, Tokyo 162-8655, Japan; Institute for Vaccine Research and Development, Hokkaido University, 5 Chome Kita 8 Jonishi, Kita Ward, Sapporo, Hokkaido 060-0808, Japan; WHO Collaborating Centre for Infectious Disease Epidemiology and Control, School of Public Health, LKS Faculty of Medicine, The University of Hong Kong, Pok Fu Lam, Hong Kong Special Administrative Region; Seattle Children’s Research Institute, 4800 Sand Point Way NE, Seattle, WA 98105, USA; Department of Pediatrics, University of Washington, 4800 Sand Point Way NE, Seattle, WA 98105, USA; WHO Collaborating Centre for Infectious Disease Epidemiology and Control, School of Public Health, LKS Faculty of Medicine, The University of Hong Kong, Pok Fu Lam, Hong Kong Special Administrative Region; Worldwide Influenza Centre, The Francis Crick Institute, 1 Midland Rd, London NW1 1AT, United Kingdom; WHO Collaborating Centre for Reference and Research on Influenza, The Peter Doherty Institute for Infection and Immunity, 792 Elizabeth St, Melbourne VIC 3000, Australia; Influenza Research Center, National Institute of Infectious Diseases, Japan Institute for Health Security, 1-21-1 Toyama Shinjuku-ku, Tokyo 162-8655, Japan; Centre for Pathogen Evolution, Department of Zoology, University of Cambridge, The Old Schools, Trinity Ln, Cambridge CB2 1TN, United Kingdom; Vaccine and Infectious Disease Division, Fred Hutch Cancer Center, 1100 Fairview Ave N, Seattle, WA 98109, USA; Howard Hughes Medical Institute, 1100 Fairview Ave N, Seattle, WA 98109, USA; Division of Basic Sciences and Computational Biology Program, Fred Hutch Cancer Center, 1100 Fairview Ave N, Seattle, WA 98109, USA; Department of Genome Sciences, University of Washington, 1959 NE Pacific St, Seattle, WA 98195, USA; Howard Hughes Medical Institute, 1100 Fairview Ave N, Seattle, WA 98109, USA

**Keywords:** influenza, antigenic drift, vaccine strain selection, sequencing-based neutralization assay

## Abstract

The hemagglutinin of human influenza virus evolves rapidly to erode neutralizing antibody immunity. Twice per year, new vaccine strains are selected with the goal of providing maximum protection against the viruses that will be circulating when the vaccine is administered ~8–12 months in the future. To help inform this selection, here we quantify how the antibodies in recently collected human sera neutralize viruses with hemagglutinins from contemporary influenza strains. Specifically, we use a high-throughput sequencing-based neutralization assay to measure how 188 human sera collected from Oct 2024 to April 2025 neutralize 140 viruses representative of the H3N2 and H1N1 strains circulating in humans as of the summer of 2025. This data set, which encompasses 26 148 neutralization titre measurements, provides a detailed portrait of the current human neutralizing antibody landscape to influenza A virus. The full data set and accompanying visualizations are available for use in vaccine development and viral forecasting.

## 1. Introduction

The antigenic evolution of human influenza virus erodes the effectiveness of pre-existing immunity from prior infections and vaccinations and is a major reason that the typical person is infected with the influenza A virus roughly every 5 years (Kucharski et al. 2015, Ranjeva et al. 2019). The most rapidly evolving viral protein is hemagglutinin (HA); this protein is the major target of neutralizing antibodies, which are a strong correlate of protection against infection (Hobson et al. 1972, Ohmit et al. 2011, Krammer 2019). The HAs of human H3N2 and H1N1 influenza acquire an average of three to four and two to three amino-acid substitutions per year, respectively (Smith et al. 2004, Bedford et al. 2014, Bedford et al. 2015).

Because of this rapid evolution, influenza vaccines are updated biannually with the aim of ensuring that they elicit neutralizing antibodies that protect well against current viral strains. Each update recommends a potentially new H3N2, H1N1, and influenza B strain (s), depending on whether there is deemed to have been substantial antigenic change in circulating viruses relative to the strains in the prior vaccine. Recommendations are made twice per year: in September for the vaccine to be administered in the next Southern Hemisphere influenza season and in February for the vaccine to be administered in the next Northern Hemisphere influenza season. Historically, recommendations were based primarily on antigenic measurements made using sera from previously naive ferrets infected with defined influenza strains (Smith et al. 2004, Jorquera et al. 2019). However, there is growing recognition that the antibodies produced by humans with extensive lifetime exposure histories can differ from those produced by singly immunized ferrets (Fonville et al. 2014, Linderman et al. 2014, Cobey and Hensley 2017, Lee et al. 2019), so antigenic measurements made using human sera are now also increasingly considered (Ampofo et al. 2015, Fonville et al. 2016, World Health Organization 2024). A variety of evolutionary forecasting approaches are used to attempt to predict which strains might dominate in the coming season (Łuksza and Lässig 2014, Neher et al. 2016, Huddleston et al. 2020, Shi et al. 2025). However, there remains a variable track record of choosing vaccine strains that turn out to be well matched to the strains that actually circulate the next season, and there is evidence that vaccine effectiveness is higher when the match is better (Gupta et al. 2006, Belongia et al. 2016, Flannery et al. 2016, Petrie et al. 2016, Lee et al. 2024).

We recently developed a sequencing-based neutralization assay that enables simultaneous measurement of neutralization to many influenza strains (Loes et al. 2024, Kikawa et al. 2025). The key innovation is to add unique nucleotide barcodes to each strain so that >100 viruses with different HAs can be pooled and assayed together, thereby enabling each column of a 96-well plate to measure the neutralization landscape of a serum against HAs from a wide diversity of strains. The neutralization titres measured using this sequencing-based assay are extremely similar to those measured using traditional one-virus versus one-serum neutralization assays (Loes et al. 2024). We have shown that at least in 2023, the actual spread of different human H3N2 influenza strains in the human population was highly correlated with the fraction of individuals with low titres against those strains as measured by this assay (Kikawa et al. 2025).

Here, we designed a library of barcoded viruses that contained HAs from human H3N2 and H1N1 influenza strains that circulated from April to May of 2025; this library continues to cover the HA diversity of these subtypes as of late summer 2025. We then used this library to measure neutralization titres for a set of 188 human sera collected from individuals of a wide range of ages at several geographic locations between October 2024 and April 2025. The resulting dataset of 26 148 neutralization titres provides a near real-time portrait of the human population’s neutralizing antibody landscape against the influenza virus that can be used to help inform vaccine strain selection in September 2025 for influenza vaccines to be used in the 2026 Southern Hemisphere season.

## 2. Methods

### 2.1. Data and code availability

See the GitHub repository at https://github.com/jbloomlab/flu-seqneut-2025 for all data and analysis code, including final neutralization titres, barcode counts of each variant in each experiment, plots of all individual neutralization curves, and detailed serum metadata. That GitHub repository includes all details; key summary files are as follows:

- Information on the tested human sera: Supplementary File 1 and https://github.com/jbloomlab/flu-seqneut-2025/blob/main/results/aggregated_analyses/human_sera_metadata.csv
- Information on the tested virus strains: Supplementary File 2 and https://github.com/jbloomlab/flu-seqneut-2025/blob/main/data/viral_libraries/flu-seqneut-2025-barcode-to-strain_actual.csv
- All measured neutralization titres after quality control: Supplementary File 3 and https://github.com/jbloomlab/flu-seqneut-2025/blob/main/results/aggregated_analyses/human_sera_titers.csv
- Summary statistics on virus-specific titres: https://github.com/jbloomlab/flu-seqneut-2025/blob/main/results/aggregated_analyses/human_sera_titers_summarized.csv
- Interactive page with links to the all neutralization curves and notebooks showing per-plate and per-serum quality-control: https://jbloomlab.github.io/flu-seqneut-2025/

### 2.2. Biosafety

All experiments utilized influenza virions with HA ectodomain proteins matching those from recent naturally occurring human seasonal H3N2 or H1N1 influenza strains. Such strains are classified as biosafety-level-2 according to the CDC BMBL handbook (edition 6). The non-HA genes were derived from the lab-adapted A/WSN/1933 (H1N1) strain, which is also classified as biosafety-level-2 according to the CDC BMBL handbook. All experimental work involving the viruses or human sera were performed at biosafety-level-2.

### 2.3. Human sera

Serum samples were sourced from individuals across ages and geographical locations through a combination of residual blood draws from hospitals, epidemiological studies, and vaccination cohorts.

Deidentified paediatric sera were obtained from children who were not immunocompromised and were undergoing routine medical care at Seattle Children’s Hospital (SCH) in April 2025 with approval from the SCH Institutional Review Board with a waiver of consent.

The deidentified remnant sera from the University of Washington (UW) Medical Center were obtained from individuals testing positive for HBsAb (to control for intact immunity) in March 2025 and was approved by the UW Institutional Review Board with a consent waiver.

The EPI-HK sera were taken from the ‘Evaluating Population Immunity in Hong Kong’ study, a community-based longitudinal observational cohort study (Cowling et al. 2022) of approximately 2000 participants of all ages run by the University of Hong Kong since 2020, and for this analysis, a subset of 42 cross-sectional sera meant to be representative of this population-based cohort at the end of the local 2024/25 Northern Hemisphere winter influenza season were selected by randomly selecting three sera from each 5-year age band between 10 and 79 years of age collected in March–April 2025. The EPI-HK study protocol was approved by the Institutional Review Board of the University of Hong Kong, and written informed consent was obtained from all study participants or their legal guardians.

The sera from the National Institute of Infectious Diseases (NIID) were from participants in a vaccine study by the NIID in Japan and included 48 sera taken from 48 unique individuals prevaccination (October–November 2024) and also 7 sera taken from 7 of these same individuals postvaccination (November–December 2024) timepoints.

All sera were treated with receptor-destroying enzyme and heat-inactivated prior to use in neutralization assays as described previously (Zost et al. 2017, Kikawa et al. 2025) in order to eliminate both sialic-acid-containing compounds that might nonspecifically inhibit viral infection. Briefly, lyophilized receptor-destroying enzyme II (Seikan) was resuspended in 20 ml phosphate buffered saline (PBS) and vacuum-filtered through a 0.22 μM filter. Then, 25 μl of sera was incubated with 75 μl of receptor-destroying enzyme (constituting a 1:4 dilution) at 37°C for 2.5 h and then 55°C for 30 min. Sera were used immediately or stored at −80°C.

### 2.4. Design of sequencing-based neutralization assay library

The goal of our library design was to choose HAs from sequenced human strains that were either at high frequency or had mutations that we deemed likely to be of antigenic or evolutionary significance. To select these 2025-circulating strains that we hoped would be representative of future HA diversity, we identified human seasonal H1N1 and H3N2 haplotypes that had been sequenced within a 6-month time period of library design (May 2025), pared down the list of recent haplotypes to only those that had a high local branching index (Neher et al. 2014) on Nextstrain (Hadfield et al. 2018) 6-month builds and/or contained mutations at antigenic sites. For H3N2 strains, we used an analysis approach conceptually similar to that previously described for SARS-CoV-2 (Bloom and Neher 2023) to identify HA mutations that have recently independently arisen more than expected from the underlying mutation rate and so are putatively beneficial to the virus and also selected representative strains containing such mutations. Altogether, this process selected 77 H3N2 strains and 39 H1N1 strains. The code for choosing strains is available at https://github.com/jbloomlab/flu-seqneut-2025/tree/main/non-pipeline_analyses/library_design, and the nucleotide and protein sequences for the HA ectodomain for all 2025-circulating strains and recent vaccine strains in the final library are available at https://github.com/jbloomlab/flu-seqneut-2025/tree/main/results/viral_strain_seqs.

In addition, our libraries also included the HAs from the component strains of both H3N2 and H1N1 seasonal vaccine strains. For H3N2, we included cell- and egg-based vaccine strains from the 2014 vaccine to present. For H1N1, we included cell- and egg-passaged vaccine strains from the 2010 vaccine to present. For both H3N2 and H1N1 vaccine strains, some egg-passaged strains had to be dropped as they did not grow to high titres in our system, which generates viruses *via* mammalian cell lines (293T and MDCK-SIAT1-TMPRSS2 cells).

Overall, our final libraries after library generation quality control steps contained 286 barcodes covering 76 recent H3N2 strains, 38 recent H1N1 strains, 19 past H3N2 egg- or cell-produced vaccine strains, and 7 past H1N1 egg- or cell-produced vaccine strains (https://github.com/jbloomlab/flu-seqneut-2025/blob/main/data/viral_libraries/flu-seqneut-2025-barcode-to-strain_actual.csv). Note that the original designed libraries contained slightly more strains and barcodes as a few dropped out during library generation and quality control: the designed libraries included 322 barcodes covering 76 recent H3N2 strains, 39 recent H1N1 strains, 22 past H3N2 egg- or cell-produced vaccine strains, and 9 past H1N1 egg- or cell-produced vaccine strains (https://github.com/jbloomlab/flu-seqneut-2025/blob/main/data/viral_libraries/flu-seqneut-2025-barcode-to-strain_designed.csv).

### 2.5. Cloning of barcoded hemagglutinins

As described previously (Loes et al. 2024, Welsh et al. 2024, Kikawa et al. 2025), the HA genes used for our libraries consist of the noncoding regions from the lab-adapted A/WSN/1933 (H1N1) HA, the first 19 (for H3 constructs) or 20 (for H1 constructs) amino acids of the N-terminal signal peptide from the A/WSN/1933 HA, the HA ectodomain from each strain of interest, the consensus H3 transmembrane domain (for H3 constructs) or the A/WSN/1933 transmembrane domain (for H1 constructs), the cytoplasmic tail from A/WSN/1933 with synonymous recoding, a double stop codon after the end of the coding sequence, followed by a 16-nucleotide barcode, the Illumina Read 1 priming sequence, and a duplicated packaging signal from A/WSN/1933. This construct enables barcoding of HAs without disrupting viral genome packaging and provides common priming sequences that can be used for barcode amplification.

For cloning the barcoded HAs into influenza reverse genetics plasmids, we used a simplified cloning strategy as described previously (Loes et al. 2024, Kikawa et al. 2025). Briefly, barcoded constructs encoding the HA ectodomains were ordered from Twist Biosciences with sequence homology at the 5′ end of the coding sequence with the lab-adapted A/WSN/1933 (H1N1) HA signal peptide and sequence homology at the 3′-end (after the barcode) with the Illumina Read 1 sequence. We tagged each HA variant with randomly generated 16-nucleotide barcodes, specifically avoiding barcode sequences used in prior libraries (Loes et al. 2024, Kikawa et al. 2025) or beginning with GG nucleotides (we found such barcodes can sequence poorly in the sequence context of our libraries). Barcode fragments were then assembled into plasmid backbones using HiFi Assembly Mastermix (NEB) per the manufacturer’s instructions.

The plasmid backbone for both the H1N1 and H3N2 constructs was the derivative of the pHH21 uni-directional reverse genetics plasmid (Neumann et al. 1999) that we have described previously (Welsh et al. 2024, Kikawa et al. 2025); see https://github.com/dms-vep/flu_h3_hk19_dms/blob/main/library_design/plasmid_maps/2851_pHH_WSNHAflank_GFP_H3-recipient_duppac-stop.gb for a map of this plasmid backbone. This plasmid was digested with enzymes XbaI and BsmBIv2 (NEB) per the manufacturer’s instructions, and the desired fragment was obtained by gel electrophoresis and purification. Each barcoded construct was then transformed into competent cells in the Bloom lab, and individual colonies were then screened and DNA prepped by Azenta/Genewiz. Exemplar plasmid maps for a H1 and H3 strain are at https://github.com/jbloomlab/flu-seqneut-2025/blob/main/non-pipeline_analyses/library_design/plasmids/example_constructs, and the full set of all plasmid maps is at https://github.com/jbloomlab/flu-seqneut-2025/blob/main/non-pipeline_analyses/library_design/plasmids. The barcodes linked to each strain in the final library are at https://github.com/jbloomlab/flu-seqneut-2025/blob/main/data/viral_libraries/flu-seqneut-2025-barcode-to-strain_actual.csv.

### 2.6. Generation and titration of viruses carrying barcoded hemagglutinins

Barcoded viruses expressing the different library HAs were generated using reverse genetics (Neumann et al. 1999, Hoffmann et al. 2000). As described previously (Loes et al. 2024, Kikawa et al. 2025), we generate the two or three barcoded variants for each HA strain in the library by pooling barcoded plasmids encoding that particular HA prior to transfecting cells. However, the barcoded variants for each strain are generated independently. For the reverse genetics, plasmid DNA mixes were made containing 250 ng of a given strain’s HA plasmid pool (containing equal amounts of the two or three of the independently barcoded constructs) with 250 ng of each of a pHW-series bidirectional reverse genetics plasmid (Hoffmann et al. 2000) encoding each non-HA segment (PB1, PB2, PA, NA, M, NP, NS) from A/WSN/1933 (H1N1). These DNA mixtures were incubated with 100 μl of Dulbecco's Modified Eagle Medium (DMEM) and 3 μl of BioT Transfection Reagent (Bioland Scientific) per the manufacturer’s instructions and then transfected onto cocultures of 5e5 293T cells and 5e4 MDCK-SIAT1-TMPRSS2 cells that had been plated ~24 h prior in 6-well dishes in D10 media (DMEM supplemented with 10% heat-inactivated fetal bovine serum (FBS), 2 mM L-glutamine, 100U per ml penicillin, and 100 μg per ml streptomycin). At ~ 16–20 h post-transfection, media were removed, cells were washed gently with 2 ml of PBS, and then 2 ml of influenza growth media (Opti-MEM supplemented with 0.1% heat-inactivated FBS, 0.3% bovine serum albumin, 100 μg per ml of calcium chloride, 100 U per ml penicillin and 100 μg per ml streptomycin) was added. After an additional ~ 45-h incubation (~65 h post-transfection), viral supernatants were aliquoted for storage at −80°C and used to set up a single viral passage (intended to reduce carry-over plasmid DNA and increase titres). For these passages, 100 μl of each viral stock was infected onto a single well of a 6-well plate containing 4e5 MDCK-SIAT1-TMPRSS2 cells in 2 ml of influenza growth media for ~40 h, as described previously (Loes et al. 2024). Supernatants were then cleared of cell debris by centrifugation at 400*g* for 5 min before being stored at −80°C.

As in prior work (Loes et al. 2024, Kikawa et al. 2025), to determine the relative transcriptional titre of each of the passaged viruses, we made an equal-volume pool, which was serially two-fold diluted and used to infect MDCK-SIAT1 cells. At 16 h postinfection, cells were lysed and viral barcodes were sequenced as described below. We used these barcode sequencing counts to determine the relative amount of each viral strain (see https://github.com/jbloomlab/flu-seqneut-2025/blob/main/non-pipeline_analyses/library_pooling/notebooks/250716_initial_equal_volume_pool.ipynb) and then repooled so that each strain’s barcodes would be present roughly equally in the pool. This repooled, equally proportioned library stock was then serially two-fold diluted and used to infect MDCK-SIAT1 cells exactly as described above, and barcode counts were used to affirm roughly equal pooling of strains and to determine the virus dilutions where viral transcription tracked linearly with the amount of virus particles added to cells as described previously (Loes et al. 2024, Kikawa et al. 2025) (see https://github.com/jbloomlab/flu-seqneut-2025/blob/main/non-pipeline_analyses/library_pooling/notebooks/250723_balanced_repool.ipynb). Based on this analysis, for the experiments described here we chose the 1:16 dilution of the virus library stock as it was in the early part of this linear range where viral transcription is linearly correlated with viral neutralization.

### 2.7. Sequencing-based neutralization assays

The experimental setup was nearly identical to that outlined previously (Kikawa et al. 2025) with a few modifications outlined below. A detailed protocol is available on protocols.io at https://dx.doi.org/10.17504/protocols.io.kqdg3xdmpg25/v2. Sera were diluted to 1:20 (accounting for this initial 1:4 dilution from receptor-destroying enzyme treatment) in 50 μl of influenza growth media and then serially 2.3-fold diluted down columns of 96-well plate. As determined in virus library titration experiments described above, the virus library was then added to all wells at a 1:16 dilution in 50 μl, resulting in a range of serum dilutions from 1:40 to 1:13619 across each 8-well column of a 96-well plate. These virus serum-mixtures were then incubated at 37°C with 5% CO_2_ for 1 h before 1.5e5 MDCK-SIAT1 cells were added per well in a total of 50 μl of influenza growth media. After a 16-h incubation, cells were lysed and barcodes were sequenced as described previously. Briefly, barcoded RNA spike-in (prepared as described previously; Loes et al. 2024) was diluted to 2 pM in the iScript Sample Preparation Reagent (BioRad) and incubated on cells for 5 min. Lysate was then transferred to new 96-well plates for storage, and 1 μl of lysate was used in 10 μl complementary DNA (cDNA) synthesis reactions using the iScript cDNA Synthesis Kit (BioRad) per the manufacturer’s instructions. As described previously (Kikawa et al. 2025), we then amplified cDNA in two rounds of PCR. The first round of PCR added a 6-bp index, allowing us to multiplex different plates using the same dual indices (added in the second round of PCR), which helped decrease sequencing costs. For this first-round PCR, forward primers were either one of the same four forward primers described previously (Kikawa et al. 2025) or a similar new forward primer (listed below). In all cases, this forward primer was paired with the same reverse primer described previously (Loes et al. 2024). For this first round of PCR, we used 2 μl of cDNA template in 25 μl PCR reactions using KOD Polymerase Hot Start 2× Mastermix (Sigma) per the manufacturer’s instructions. The four new forward primers were:

5’-GTGACTGGAGTTCAGACGTGTGCTCTTCCGATCTgtctaaCCTACAATGTCGGATTTGTATTTAATAG-3′

5’-GTGACTGGAGTTCAGACGTGTGCTCTTCCGATCTacgctgCCTACAATGTCGGATTTGTATTTAATAG-3′

5’-GTGACTGGAGTTCAGACGTGTGCTCTTCCGATCTtatagcCCTACAATGTCGGATTTGTATTTAATAG-3′

5’-GTGACTGGAGTTCAGACGTGTGCTCTTCCGATCTcgagctCCTACAATGTCGGATTTGTATTTAATAG-3′)

In the second round of PCR, unique dual indexing primers (described previously; Loes et al. 2024) were added in 25 μl reactions, again using KOD Polymerase Hot Start 2× Mastermix (Sigma) per the manufacturer’s instructions. These second-round PCR products were then pooled at equal volume, gel-extracted, purified, quantified, and sequenced exactly as described previously (Kikawa et al. 2025).

### 2.8. Analysis of sequencing data to determine neutralization titres

The sequencing data were analysed as described previously (Loes et al. 2024, Kikawa et al. 2025) using the *seqneut-pipeline* (https://github.com/jbloomlab/seqneut-pipeline), version 4.0.1. Briefly, in this pipeline, the Illumina sequencing data are parsed to count each barcoded variant in each well of each plate (see https://github.com/jbloomlab/flu-seqneut-2025/tree/main/results/barcode_counts for these counts). The barcode counts are then normalized to fractional infectivity of each variant at each serum concentration using the counts of the RNA spike-in standard (see the ‘frac_infectivity.csv’ files for each plate at https://github.com/jbloomlab/flu-seqneut-2025/tree/main/results/plates). The *neutcurve* package (https://github.com/jbloomlab/neutcurve) package is used to fit Hill curves for each variant and serum, and the titre is quantified as the midpoint of the curves. See the analysis configuration file (https://github.com/jbloomlab/flu-seqneut-2025/blob/main/config.yml) for details about the parameters used for the curve fitting and subsequent quality control to remove low-quality curves. For HAs with multiple barcodes, we report the median titre across barcodes.

A fully reproducible *Snakemake* (Mölder et al. 2021) pipeline that performs the above analysis is available on GitHub at https://github.com/jbloomlab/flu-seqneut-2025. See https://jbloomlab.github.io/flu-seqneut-2025 for HTML rendering of all neutralization curves, quality control notebooks, and interactive plots summarizing the data.

### 2.9. Phylogenetic analyses with Nextstrain

For each subtype, we created a phylogenetic tree for the HA nucleotide sequences used in the neutralization assays along with approximately 500 additional HA sequences from the Global Initiative on Sharing All Influenza Data (GISAID) EpiFlu database (Shu and McCauley 2017) collected between 1 August 2024 and 22 August 2025 to show the genetic context of library sequences and the genetic diversity that has emerged globally since we finalized the library design. We required contextual sequences to have complete collection dates and we excluded known outliers that we previously identified through weekly genomic surveillance analyses with Nextstrain (H3N2 outliers at https://github.com/nextstrain/seasonal-flu/blob/825314f/config/h3n2/outliers.txt and H1N1pdm outliers at https://github.com/nextstrain/seasonal-flu/blob/825314f/config/h1n1pdm/outliers.txt). We randomly sampled the contextual sequences, evenly sampling from each major global region and month. We aligned sequences to a reference virus sequence (A/Wisconsin/67/2005 for H3N2 and A/Wisconsin/588/2019 and H1N1pdm) with Nextclade version 3.16.0 (Aksamentov et al. 2021) and inferred a divergence tree with IQ-TREE version 3.0.1 (Wong et al. 2025) using the augur tree (Huddleston et al. 2021). We rooted the tree with the reference virus, pruned the reference virus from the tree, and inferred a time tree with TreeTime version 0.11.4 (Sagulenko et al. 2018) using a fixed clock rate (0.00382 for H3N2 and 0.00329 for H1N1pdm), a clock standard deviation of one-fifth the clock rate, a constant coalescent, marginal date inference, stochastic polytomy resolution, and Fast Fourier Transform (FFT) inference. We inferred ancestral nucleotide and amino acid sequences for internal nodes with TreeTime through the augur ancestral command and used the mutations associated with these inferred sequences to annotate clades with augur clades. For each tree, we created a corresponding measurements panel (Lee et al. 2023) containing the log_2_ neutralization titres per serum id. These analyses are available through the reproducible *Snakemake* pipeline at https://github.com/blab/kikawa-seqneut-2025-VCM/. A complete list of GISAID accessions and authors is available at https://github.com/blab/kikawa-seqneut-2025-VCM/blob/79668f4/gisaid_accessions.tsv.

To infer phylogenetic trees from HA protein sequences such that branch length reflects the number of amino acid mutations separating different viral strain HA sequences, we developed a separate *Snakemake* pipeline and integrated our titre data with this phylogenetic analysis as well. The pipeline for building the trees is described at https://github.com/jbloomlab/nextstrain-prot-titers-tree, with the configuration for our analysis placed at https://github.com/jbloomlab/flu-seqneut-2025/blob/main/config.yml. These protein-based trees can be explored at https://nextstrain.org/community/jbloomlab/flu-seqneut-2025@main/H3N2 and https://nextstrain.org/community/jbloomlab/flu-seqneut-2025@main/H1N1.

## 3. Results

### 3.1. A library of influenza hemagglutinins that covers the antigenic diversity of human H3N2 and H1N1 influenza as of the summer of 2025

**Figure 1:**
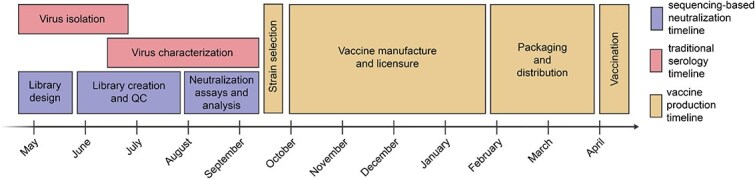
Typical timeline for data collection to inform influenza vaccine strain selection and subsequent production of vaccine for the Southern Hemisphere influenza season. Vaccine strain recommendations are made twice per year, once for the Southern Hemisphere vaccine and once for the Northern Hemisphere. The timeline above illustrates typical timeframes for influenza vaccine strain selection and production during the Southern Hemisphere influenza seasons for traditional virus isolation/characterization and vaccine production, alongside the timeframe for our sequencing-based neutralization assay measurements reported in the current study.

**Figure 2:**
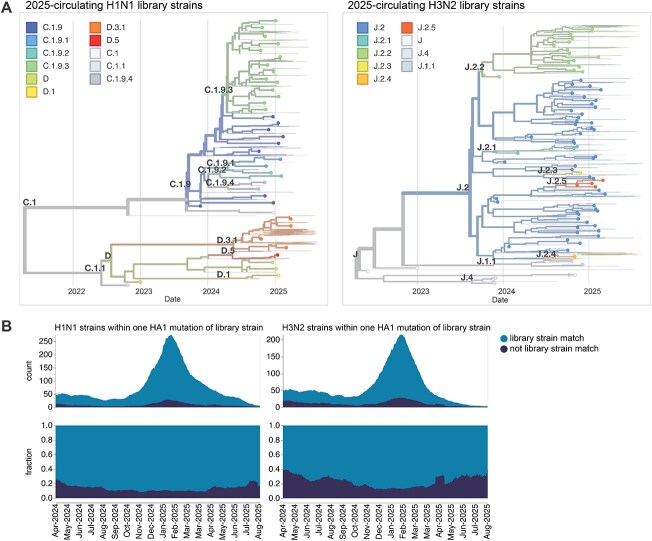
HAs included in the sequencing-based neutralization library. (a) Phylogenetic trees of HA genes of H1N1 and H3N2 strains included in the libraries. Strains included in the library are indicated with points, and other recent representative strains are shown in light lines. Colours indicate the subclade designation of each strain. These trees show only the recent strains designed to cover the current diversity of viral strains, as well as the most recent vaccine strains (vaccine strains from 2024 seasons to present for both H1N1 and H3N2); note that the libraries also contained older vaccine strains dating back to 2012 for H3N2 and 2009 for H1N1. Interactive Nextstrain versions of these trees are available at (https://nextstrain.org/groups/blab/kikawa-seqneut-2025-VCM/h1n1pdm?f_kikawa=present_1&p=grid) and (https://nextstrain.org/groups/blab/kikawa-seqneut-2025-VCM/h3n2?f_kikawa=present_1&p=grid). (b) Count and fraction of all sequenced human seasonal H1N1 and H3N2 HAs available as of 28 August 2025 that are or are not within one HA1 amino-acid mutation of a strain in our libraries. The counts and fractions shown here are computed over a 10-day sliding window over the strain collection dates.

Our goal was to design a library of HAs that could be used to measure strain-specific neutralization titres prior to the September 2025 vaccine-strain selection (Fig. 1). In April–May 2025, we chose a set of HAs to cover the genetic and antigenic diversity of human H3N2 and H1N1 strains that had been sequenced at that time. This library consisted of the HAs from 76 recently circulating human H3N2 strains and 38 recently circulating human H1N1 strains, as well as a total of 26 vaccine strains dating back to the 2014 vaccine for H3N2 and the 2010–2011 vaccine for H1N1 (Fig. 2). We chose the recently circulating strains to include high-frequency HA protein genotypes in the 6-month period prior to library design, as well as strains observed within that time period that contained mutations at sites known to be antigenically important (see Section 2 for details).

The HAs included in this library continue to effectively encompass most of the diversity of human H3N2 and H1N1 influenza as of late August 2025, with ~78% of H1N1 and ~67% of H3N2 viruses sequenced over the summer of 2025 being within a single HA1 amino-acid mutation of a strain in the library (Fig. 2).

**Figure 3:**
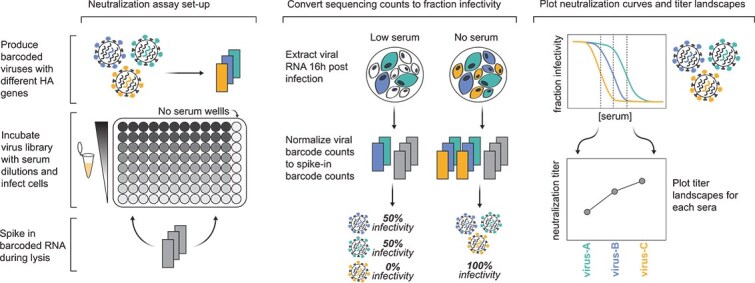
Sequencing-based neutralization assays enable rapid measurement of titres against many strains. Barcoded influenza viruses expressing different HAs are pooled and mixed with sera and MDCK-SIAT1 cells in an experimental set-up similar to traditional neutralization assays. To read out neutralization, viral RNA is extracted 16 h postinfection. A known concentration of barcoded RNA spike-in is added to each well and used to normalize viral RNA barcode counts, and these normalized counts are used to calculate the infectivity of each viral strain at each serum concentration relative to wells with no serum. Neutralization curves are fit to these percent infectivity values, and neutralization titres (defined as the reciprocal of the serum dilution at which 50% of viruses are neutralized) are calculated from these curves. The viral libraries used in this study contained viruses with 140 different HAs, and each plate was set up to assay 11 sera, meaning that each plate measured a total of 1540 titres. Most HAs are represented multiple times in the viral libraries with several distinct barcodes, meaning most titres are measured in replicate in each plate.

We individually generated barcoded viruses carrying each of the different HAs (Fig. 3) using previously described approaches (Loes et al. 2024, Kikawa et al. 2025). The HA genes were tagged with identifying 16-nucleotide barcodes and encoded the ectodomains from the naturally occurring recent H3N2 and H1N1 strains and were incorporated into virions with the other seven viral genes derived from the lab-adapted A/WSN/1933 strain. We aimed to generate two or three distinct barcoded viruses for each HA variant to provide internal replicates in the sequencing-based neutralization assays. With these barcode replicates, after quality control, we had a total of 286 unique barcoded viral variants for the 140 different HAs. To create a library pool with all the variants at roughly equal titres of transcriptionally active particles, we pooled all the variants at equal volume, infected cells, extracted RNA, used barcode sequencing to quantify the relative transcriptional contribution of each variant, and then used these data to re-pool variants to balance the transcriptionally active particle titres for all HAs.

### 3.2. A diverse collection of sera collected from humans in late 2024 and early 2025

**Figure 4:**
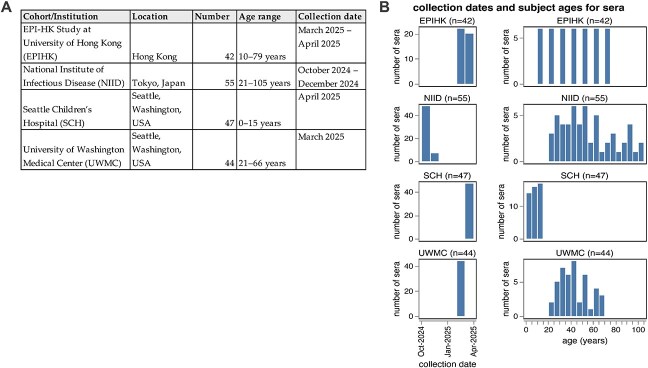
Human sera used in the neutralization assays. The human sera used in this study came from four different sources. (a) Details on the four groups of sera tested. All sera are from unique individuals with the exception of the NIID cohort; for that cohort 48 sera are from unique individuals prevaccination, and 7 sera are from 7 of those same individuals ~ 1–2 months postvaccination. (b) Distribution of collection dates and ages of individuals from which the sera were collected for each group.

We assembled 188 human sera collected from individuals spanning from young children to elderly adults and drawn from four sites around the world (Fig. 4). These sera were collected between October 2024 and April 2025. Some sera are from individuals with some information on recent vaccination and infection status; for example, in the EPI-HK study, 19/42 (45%) participants reported receipt of the 2024/25 Northern Hemisphere influenza vaccine and 1/42 (2%) had PCR-confirmed influenza virus infection identified in-house within 182 days from the date of serum collection (Supplementary File 1). However, many sera are residual samples from individuals with unknown infection and vaccination histories. Because the influenza immunity of most humans is due to a combination of infection and vaccination (with many individuals not receiving vaccines), and because infection often leaves a more durable imprint on antibody titres (Davis et al. 2020, Turner et al. 2020) than vaccination, assaying residual sera from individuals with unknown vaccination histories as well as sera from well-characterized cohorts helps capture the diversity of anti-influenza serum antibodies across the human population.

### 3.3. Human neutralizing antibody landscape against recent H3N2 and H1N1 strains

We used the sequencing-based neutralization assay (Fig. 3) to measure neutralization curves for the 140 viruses against all 188 sera. After quality control to remove low-quality neutralization curves, we had a set of 26 148 neutralization titres (we quantify the neutralization titre as the reciprocal serum dilution that neutralizes 50% of the infectivity of a given viral strain as measured by our sequencing-based approach). Here, we summarize major trends relevant to characterizing the neutralizing antibody landscape against the full set of viral strains across the tested sera; Section 2 provides links to the full numerical titre data.

**Figure 5:**
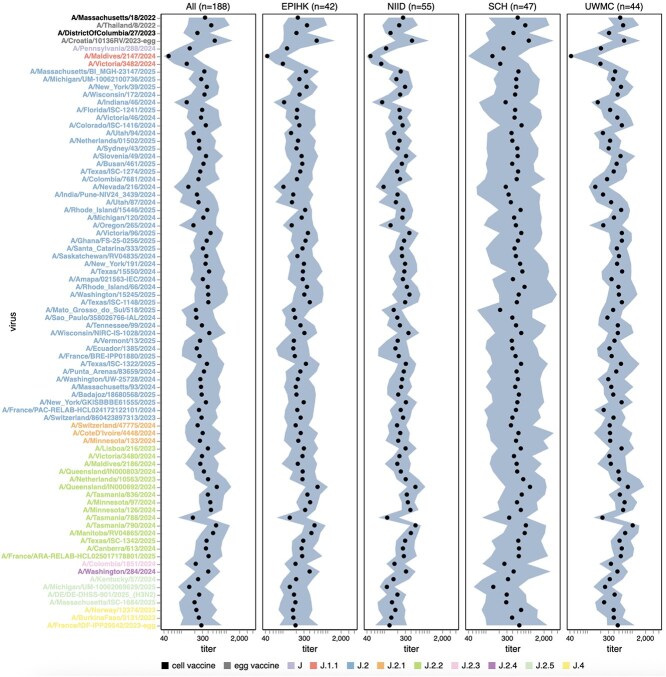
Human neutralizing antibody landscape against recent H3N2 strains. Median (black points) and interquartile range (shaded area) of the titres of the sera against the 76 H3N2 strains that capture current circulating diversity, as well as the last two sets of cell- and egg-produced vaccine strains. The leftmost plot shows the titres against all sera, and the remaining plots show titres for sera from each different cohort. Strain labels are coloured by whether a strain is a cell- or egg-produced vaccine strain or its subclade per the legend at bottom. The lower limit of detection for titres in our neutralization assays was 40. See https://jbloomlab.github.io/flu-seqneut-2025/human_sera_titers_H3N2_recent_interquartile_range.html for an interactive version of this plot that allows mousing over points for details about individual viruses, and subsetting on sera from specific age ranges.

**Figure 6:**
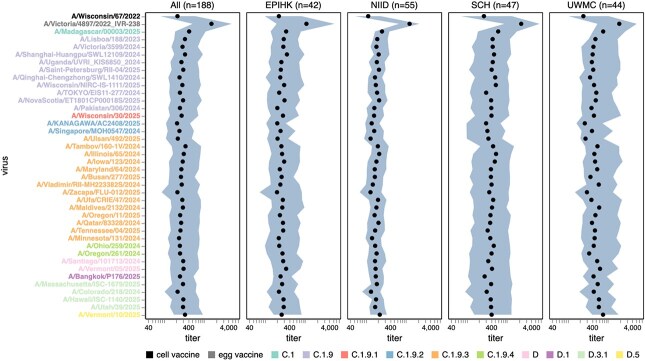
Human neutralizing antibody landscape against recent H1N1 strains. Median (black points) and interquartile range (shaded area) of the titres of the sera against the 38 H1N1 strains that capture current circulating diversity, as well as the last set of cell- and egg-produced vaccine strains. The leftmost plot shows the titres against all sera, and the remaining plots show titres for sera from each different cohort. Strain labels are coloured by whether a strain is a cell- or egg-produced vaccine strain or its subclade per the legend at the bottom. The lower limit of detection for titres in our neutralization assays was 40. See https://jbloomlab.github.io/flu-seqneut-2025/human_sera_titres_H1N1_recent_interquartile_range.html for an interactive version of this plot that allows mousing over points for details about individual viruses, and subsetting on sera from specific age ranges.

**Figure 7:**
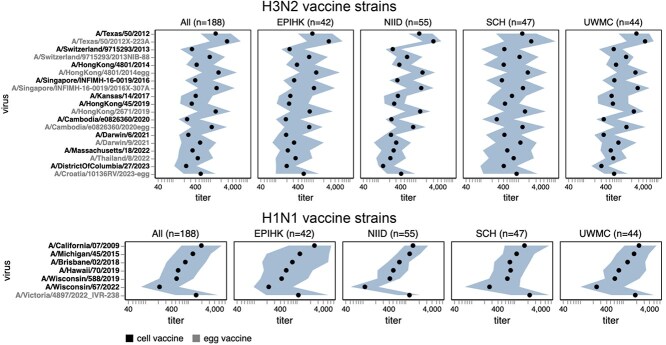
Human neutralizing antibody landscape against past vaccine strains. Median (black points) and interquartile range (shaded area) of the titres of the sera against past H3N2 (top) and H1N1 (bottom) vaccine strains, showing both the cell- and egg-produced vaccine strains for most seasons. The leftmost plot shows the titres against all sera, and the remaining plots show titres for sera from each different cohort. Strain labels are coloured by whether a strain is a cell- or egg-produced vaccine strain per the legend at the bottom. The lower limit of detection for titres in our neutralization assays was 40. See https://jbloomlab.github.io/flu-seqneut-2025/human_sera_titres_H3N2_vaccine_interquartile_range.html and https://jbloomlab.github.io/flu-seqneut-2025/human_sera_titres_H1N1_vaccine_interquartile_range.html for an interactive version of this plot that allows mousing over points for details about individual viruses, and subsetting on sera from specific age ranges.

The neutralization titres were highly heterogeneous both across sera from different individuals and across viral strains (Supplementary Figs S1–S3). Some of this heterogeneity was due to wide serum-to-serum variation in titres against all strains, as some individuals have generally higher anti-influenza neutralizing antibody titres than others. The heterogeneity in titres across sera was especially striking for children (e.g. see the Seattle Children’s Hospital Cohort, SCH, in Supplementary Figs S1–S3), consistent with prior work (Welsh et al. 2024, Kikawa et al. 2025). To summarize the variation in titres due to differences between viral strains, we computed the median and interquartile range of the neutralizing titres against each viral strain across all sera (Figs 5–7).

Among the recent H3N2 strains that capture current viral diversity, the median titres against different strains varied by more than five-fold (Fig. 5 and Supplementary Fig. S1). The titres against the most recent (2025–2026) cell-produced H3N2 vaccine strain A/DistrictOfColumbia/27/2023 are comparable to those against many other strains in the library, suggesting that most currently circulating strains are not substantially antigenically advanced compared to this vaccine strain. However, some strains scattered across several subclades are neutralized by typical human sera substantially less well than this vaccine strain. The lowest median titres are to the J.1.1 subclade strain A/Maldives/2147/2024, and titres are also low to the related J.1.1 subclade strain A/Victoria/3482/2024; these strains share HA antigenic mutations I25V, S145N, and I214T. However, the J.1.1 subclade has only been observed at low frequency recently, possibly suggesting other factors may limit its spread. Across multiple subclades, strains containing mutations at site 158 (e.g. the J.2.5 strain A/Massachusetts/ISC-1684/2025) or both sites 158 and 189 (e.g. the J.2.3 strain A/Colombia/1851/2024, the J.2 strain A/Mato_Grosso_do_Sul/18/2025, and the J.2.5 strain A/Michigan/UM-10062069269/2025) also tend to be neutralized poorly relative to other strains for some sera. Other strains that have relatively lower neutralization titres for some sera include the J.2.2 strain A/Tasmania/788/2024, and the J.2 strains A/Nevada/216/2024, A/Oregon/265/2024, and A/Indiana/46/2024. The same strains mentioned above with low median titres also tend to be ones with the highest fraction of sera with titres below a cutoff of 140 (Supplementary Fig. S4), a feature that we previously showed correlated with strain evolutionary success in 2023 (Kikawa et al. 2025).

Among the recent H1N1 strains, the median titres were less variable across strains than for H3N2, with only about two-fold variation in median titres among strains (Fig. 6 and Supplementary Fig. S2). The titres against the most recent (2025–2026) cell-produced H1N1 vaccine strain (A/Wisconsin/67/2022) were actually lower than those against most (but not all) other strains in the library. The titres against the most-recent egg-produced H1N1 vaccine strain (A/Victoria/4897/2022) were substantially higher than those against any recent circulating H1N1 strain, possibly because this strain contains several egg-adaptation mutations (Zost et al. 2017, Garretson et al. 2018), including the R142K reversion and Q223R relative to the cell-produced vaccine strain (Suptawiwat et al. 2013, Skowronski et al. 2024). Although no recent strains have a substantially lower median titre than others, there are a few strains in different subclades that do have substantial reductions in titre for subsets of sera, including the C.1.9.2 subclade strain A/KANAGAWA/AC2408/2025 and the C.1.9.3 subclade strain A/Ulsan/492/2025 (Supplementary Fig. S2). Both of these strains contain the G155E mutation, which can arise during lab-passaging of H1N1 (Guarnaccia et al. 2013) but is also sporadically observed in strains with no reported lab passaging, albeit only at low frequency among current strains. There was some modest variation across recent H1N1 strains in the fraction of sera that fall below a titre cutoff of 140, but this variation was much less than for recent H3N2 strains (compare Supplementary Figs S4 and S5).

### 3.4. Neutralizing antibody landscape to past vaccine strains

The library also contained past vaccine strains to complement the strains reflecting the current diversity of H3N2 and H1N1 seasonal influenza. Here, we summarize notable trends in the titres of the human sera against these vaccine strains.

In recent years, separate strains have been chosen for vaccines produced in either cells or eggs, since human seasonal strains often do not grow well in eggs without adaptive mutations in HA (Zost et al. 2017, Garretson et al. 2018). The median titres to both H1N1 and H3N2 historical vaccine strains were consistently higher against the egg-produced vaccine strains relative to their cell-produced counterparts chosen for the same seasons, with titres to some pairs of egg- versus cell-produced vaccine strains for the same season differing by more than five-fold (Fig. 7 and Supplementary Fig. S3). This trend could be due to the antigenic effects of HA mutations selected by egg-passaging (Zost et al. 2017, Garretson et al. 2018, Skowronski et al. 2024) or reduced receptor avidity (Hensley et al. 2009) of egg-passaged viruses (the egg-produced viruses tended to be lower titre than the cell-produced equivalents on the MDCK-SIAT1 cells used in our experiments, favouring the latter hypothesis). For the H3N2 vaccine strains, the difference in titres between egg- and cell-passaged strains was lessened for more recent strains (Fig. 7 and Supplementary Fig. S3).

Most sera had higher titres to older *versus* newer vaccine strains (Fig. 7 and Supplementary Fig. S3), a result that makes sense as most sera in our study are from adults and extensive prior work has established that immune imprinting and back-boosting mean that humans tend to have higher titres to strains encountered earlier in their lives (Lessler et al. 2012, Fonville et al. 2014, Ranjeva et al. 2019). However, this trend is lessened or even reversed in the two cohorts that include children (SCH and EPI-HK), a fact that is most easily seen by using the sliders in the interactive versions of Fig. 7 and Supplementary Fig. S3 linked in their legends to subset just on sera from children—perhaps because the older vaccine strains only circulated before these children were born.

### 3.5. Interactive visualization of neutralization titres in a phylogenetic context

**Figure 8:**
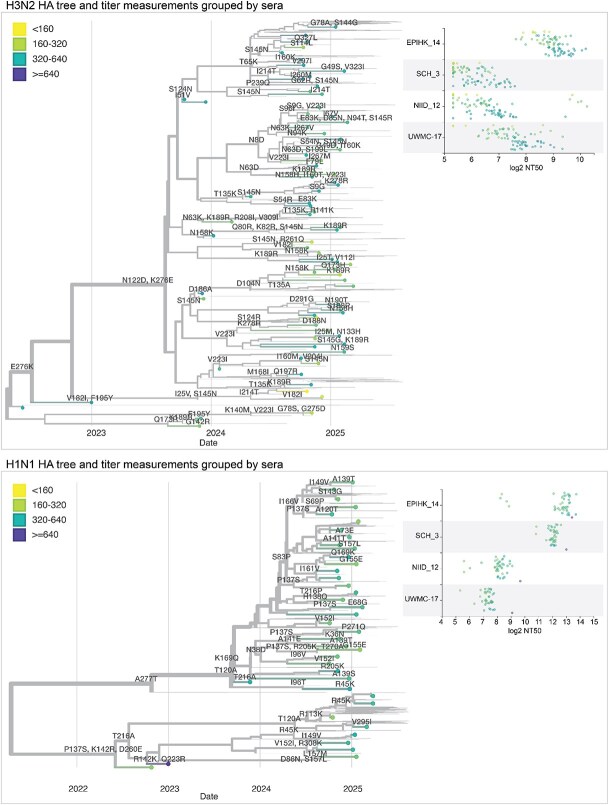
Static views of interactive Nextstrain trees showing the neutralization titres shown on HA phylogenetic trees. Phylogenetic trees of the HA gene of human H3N2 or H1N1 influenza, showing recent strains in the libraries as points, with thin lines representing other recent sequences. Circles representing the tips are coloured by the median neutralization titre across all sera as measured in the current study. HA mutations are labelled on branches. The panels at right show the titres for four example sera across all viruses in the library. See https://nextstrain.org/groups/blab/kikawa-seqneut-2025-VCM/h3n2?c=median_titre&f_kikawa=present_1 and https://nextstrain.org/groups/blab/kikawa-seqneut-2025-VCM/h1n1pdm?c=median_titre&f_kikawa=present_1 for interactive Nextstrain versions of these nucleotide trees. Code to generate these nucleotide trees is available at https://github.com/blab/kikawa-seqneut-2025-VCM/. We also generated protein sequence-based trees (where branch lengths are in units of amino-acid mutations) for only the HAs in the sequencing-based neutralization assay libraries that can be viewed at https://nextstrain.org/community/jbloomlab/flu-seqneut-2025@main/H3N2 and https://nextstrain.org/community/jbloomlab/flu-seqneut-2025@main/H1N1.

We integrated our neutralization titre dataset into interactive visualizations of each subtype’s HA phylogeny using Nextstrain (Hadfield et al. 2018) (Fig. 8 and links to interactive trees in the figure legend). These trees show the phylogenetic relationships among HA sequences alongside a measurements panel where neutralization titres can be simultaneously visualized and compared to the tree (Lee et al. 2023). The phylogeny and measurements panel are linked such that filtering or colouring the tree simultaneously filters or colours the titre measurements.

We coloured tree tips by median neutralization titres across all sera, annotated the tree with HA1 amino-acid mutations, and used the measurements panel to examine titres for specific viral strains and sera (Fig. 8). For instance, the phylogeny shows how a subset of H3N2 strains with mutations 158 K and 189R have lower median titres; further interactive examination of the tree shows that those viruses are in the J.2.5 subclade (Fig. 8). The corresponding measurements panel shows how there is substantial variation across individual sera.

The interactive trees also make it possible to explore the titre data in the context of specific clades or viral mutations. For example, colouring the H3N2 tree by amino-acid identity at HA site 158 makes it easy to see how some sera have neutralization titres that are markedly affected by mutations at that site (Supplementary Fig. S6).

## 4. Discussion

We have used a sequencing-based neutralization assay to measure the neutralization of 140 strains covering the current diversity of human influenza A virus by the antibodies in 188 human serum samples taken from individuals of a wide range of ages and geographies. Our results reveal substantial variation in titres both across sera from different individuals and across different viral strains for sera from the same individuals. The heterogeneous antibody landscape captured by our measurements will shape both the distribution of infections and the evolutionary dynamics of seasonal influenza over the coming year, as there is clear evidence that humans are more likely to be infected with strains to which they have low neutralizing antibody titres (Hobson et al. 1972, Ohmit et al. 2011, Belongia et al. 2016, Flannery et al. 2016, Petrie et al. 2016, Krammer 2019, Kim et al. 2024).

Here we have mostly examined the titres after aggregation across sera from many different individuals. Some of the serum-specific patterns in the antibody landscapes are likely shaped by vaccination and infection history (Fonville et al. 2014, Cobey and Hensley 2017), and so there is ample room for future analysis of the data in terms of subject age and the available information about recent vaccinations and exposures. Additionally, our study uses sera from individuals of a wide range of ages from several geographic locations (albeit all in the Northern Hemisphere)—but an important area for future work is to determine the role that the antibody immunity of different subsets of the global population play in shaping influenza evolution and transmission (Wallinga et al. 2006, Basta et al. 2009, Kim et al. 2024, Welsh et al. 2024).

The measurements reported here represent the largest-ever single-study dataset of influenza neutralizing antibody titres, with the entire effort being completed in less than 6 months—with the experimental work performed nearly entirely by a single person. The fact that so much titre data can be generated so rapidly demonstrates how new experimental techniques enable near real-time measurement of the human neutralizing antibody landscape to influenza, potentially enabling the use of such data in immediate public-health applications in addition to retrospective studies.

Here, we have made an intentional decision to report the dataset immediately with just simple visualizations, rather than sharing the data only after lengthy post hoc analyses designed to draw final conclusions. The reason is that these data were generated with the goal of helping inform influenza vaccine-strain selection in September 2025 for the 2026 Southern Hemisphere vaccine; sharing these data immediately facilitates further analysis and interpretation by scientists involved in vaccine-strain selection as well as the larger scientific community. After the completion of this study and posting of the initial version as a *bioRxiv* preprint, these data were used as one of many sources to help inform vaccine strain selection in September 2025 (Huddleston et al. 2025). The influenza A components were both updated; the H1N1 component was updated to a D.3.1 strain (Missouri/11/2025 in both egg- and cell-based formulations), while the H3N2 component was updated to a J.2.4 strain (A/Singapore/GP20238/2024 in the egg-based formulation and A/Sydney/1359/2024 in the cell-based formulation) (‘Recommended Composition of Influenza Virus Vaccines for Use in the 2026 Southern Hemisphere Influenza Season’, n.d.). The H1N1 component strain selected for the vaccine included a 113 K mutation that analysis of our large-scale neutralization dataset reported here showed caused measurable antigenic change (Huddleston et al. 2025).

It will only be possible to determine in retrospect the extent to which the data presented here are informative for determining which viral strains or mutations will spread in the human population or indeed how well the Southern Hemisphere vaccine components will be matched to the circulating variants in 2026. It also remains unclear exactly how the large dataset we report here can best be incorporated into quantitative models (Łuksza and Lässig 2014, Neher et al. 2016, Huddleston et al. 2020, Shi et al. 2025) of how immune pressure shapes influenza virus evolution, as it is important to remember that antigenic change is only one component of viral fitness. Here, we have freely shared the dataset so both ourselves and other scientists can leverage it to advance basic and applied goals related to understanding influenza virus evolution and immunity.

## Supplementary Material

File_S1_veaf086

File_S2_veaf086

File_S3_veaf086

supplementary-material_veaf086

## Notes

Conflict of interest: J.D.B. consults for Apriori Bio, Invivyd, GlaxoSmithKline, Pfizer, and the Vaccine Company. J.D.B. and A.N.L. are inventors on Fred Hutch–licensed patents related to high-throughput viral serological assays. B.J.C. has consulted for AstraZeneca, Fosun Pharma, GlaxoSmithKline, Haleon, Moderna, Novavax, Pfizer, Roche, and Sanofi Pasteur. A.L.G. reports contract testing to UW from Abbott, Cepheid, Novavax, Pfizer, Janssen, and Hologic; research support from Gilead; and personal fees from Arisan Therapeutics, outside of the described work.
